# The impact of genetic variants in the *CYP2C8* gene on bladder cancer susceptibility

**DOI:** 10.3389/fendo.2022.989030

**Published:** 2022-09-28

**Authors:** Weixing Qu, Fuzhou Zhang, Yongyi Cheng, Jing Li, Jiancheng Zhou

**Affiliations:** ^1^ Department of Urology, Shaanxi Provincial People’s Hospital, Xi’an, China; ^2^ Department of Urology, First Hospital of Weinan City, Weinan, China

**Keywords:** *CYP2C8*, genetic variants, susceptibility, bladder cancer, case–control study

## Abstract

**Background:**

Bladder cancer is the most common leading cause of mortality around the world. Previous studies have indicated that genetic factors are significantly associated with bladder cancer progression—for instance, the *CYP2C8* gene is involved in bladder cancer progression. However, little is known about the impact of *CYP2C8* genetic polymorphisms on bladder cancer risk. We aimed to detect the association between *CYP2C8* variations and bladder cancer susceptibility.

**Methods:**

This study included 550 healthy subjects and 217 bladder cancer patients. The odds ratios (ORs) and 95% confidence intervals (CIs) were calculated to determine the correlation of *CYP2C8* polymorphisms with bladder cancer risk. Multifactor dimensionality reduction (MDR) was carried out to investigate the influence of single-nucleotide polymorphism (SNP)–SNP interactions on bladder cancer.

**Results:**

Our study showed that two SNPs were significantly associated with an increased risk of bladder cancer (rs1934951: OR 1.96, 95% CI 1.37–2.82, *p* = 2.67E-04; rs17110453: OR 1.89, 95% CI 1.35–2.67, *p* = 2.53E-04). On the contrary, two SNPs identified in the study had protective effects on bladder cancer (rs1934953: OR 0.26, 95% CI 0.14–0.47, *p* = 1.20E-05; rs2275620: OR 0.40, 95% CI 0.21–0.76, *p* = 0.005). The MDR analysis suggested that the combination of rs1934953, rs1934951, rs2275620, and rs17110453 was the best model to predict bladder cancer (CVC 10/10, testing accuracy 0.6720, *p* < 0.0001).

**Conclusion:**

There was a significant association between *CYP2C8* polymorphisms (rs1934953, rs1934951, rs2275620, and rs17110453) and susceptibility to bladder cancer.

## Introduction

Bladder cancer is one of the most common tumors in the urinary system all over the world (1, 2), ranking as the seventh and sixth leading cause of mortality and morbidity among women and men, respectively, with approximately 199,922 deaths and 549,393 new diagnoses in 2018 worldwide (2). The incidence of bladder cancer in men is three to four times higher than that in women, and the incidence in both men and women increases with age (3). Bladder cancer is a complex and multifactorial disease affected by some risk factors such as sex, age, tobacco smoking, environmental pollution, chemical carcinogen exposure, and lifestyle (4–8). However, not all individuals exposed to risk factors develop bladder cancer, indicating that individual genetic diversity plays a crucial role in bladder cancer occurrence. Moreover, an increasing number of studies have revealed that genetic factors have become one of the most important factors in the pathogenesis of bladder cancer (9, 10). The molecular mechanism of bladder cancer is mainly due to exogenous metabolic changes and mutations in genes related to DNA repair, cell proliferation, and tumor inhibition (11–13). Current evidence have suggested that genetic polymorphisms are significantly correlated with the development of bladder cancer (14–17). The study of genetic polymorphisms has enhanced our understanding of the pathogenesis of bladder cancer, so it is of great significance to find more genetic risk factors.

Single-nucleotide polymorphism (SNP) is not only the most common genetic diversity but also a new genetic biomarker, which can affect the gene regulation function by changing gene sequences, ultimately resulting in the alteration of its functional properties. A growing number of SNPs are observed to be related to bladder cancer (18). Cytochrome P450 2C8 (*CYP2C8*) is a member of the human *CYP2C* enzyme family. It has been certified that *CYP2C8* is involved in the metabolism of many exogenous compounds (19). *CYP2C8* is highly expressed in human liver, and it also can be detected in the duodenum, ovary, heart, kidney, and mammary gland (20, 21). The abnormal expression of the *CYP2C8* gene is involved in the progression of many human cancers, such as hepatocellular carcinoma, breast cancer, prostate cancer, and endometrial tumor (22, 23). We noticed that *CYP2C8* showed a significantly higher expression in bladder urothelial carcinoma compared with that in normal tissue (http://ualcan.path.uab.edu/cgi-bin/TCGAExResultNew2.pl?genenam=CYP2C8&ctype=BLCA). Taken together, we speculated that the polymorphisms of the *CYP2C8* gene play a potential role in bladder cancer development.

To our knowledge, there is no study focusing on the association between *CYP2C8* polymorphisms and bladder cancer risk. Therefore, our present study was performed to investigate whether the genetic polymorphisms (rs1934953, rs1934951, rs2275620, and rs17110453) in the *CYP2C8* gene can affect the bladder cancer susceptibility in the Chinese population.

## Materials and methods

### Study subjects

This case–control study included 217 bladder cancer patients and 550 unrelated healthy subjects admitted to the Shaanxi Provincial Cancer Hospital. We informed each subject about the purpose of the study and obtained informed consent from all participants before conducting our research. This study was approved by the Ethics Committees of Shaanxi Provincial Cancer Hospital (no. 2017SF-152). All procedures performed in the study were in accordance with the Helsinki Declaration. The case group must meet the following inclusion criteria: (1) patients with newly diagnosed, histologically confirmed bladder cancer; (2) patients with age from 18 to 80 years; (3) no preoperative chemoradiotherapy was performed; and (4) no other tumors. The exclusion criteria for all patients were as follows: (1) previous diagnosis of any cancer, metastasized cancer, and serum prostate-specific antigen (>2.5 ng/ml); (2) a family history of cancers, including bladder cancer; (3) previous chemotherapy, radiotherapy, or radical cystectomy; and (4) those with bladder tumors secondary to other malignancies. The control subjects were healthy people who have physical examinations at the same hospital with cases. The inclusion criteria for the controls were as follows: (1) healthy controls were genetically unrelated subjects and were matched to cases on age and gender; (2) there was no gross or microscopic hematuria; and (3) the ultrasonography of the bladder was normal. The controls with a previous malignancy, metastasized cancer from other or unknown origin, and family history of cancers and familial or genetic diseases were excluded. Subjects with any degree of hematuria, benign prostate hyperplasia, urinary symptoms, history of prostatitis, and pre-cancerous lesions were excluded from the study. Demographic and pathological data including gender, age, and clinical stage were obtained from the participants’ medical records. A family history of bladder cancer was considered positive when a first- or second-degree relative of the participants was diagnosed with bladder cancer. None of the individuals included in this study was under occupational exposure to hazardous carcinogens related to bladder cancer.

### SNP selection and genotyping

The detailed steps of SNP selection are as follows: (1) We obtained the physical position of the *CYP2C8* gene on chromosome 10: 95,036,772–95,069,497 through the human e!GRCh37 database (http://asia.ensembl.org/Homo_sapiens/Info/Index). In the VCF to PED Converter window (http://grch37.ensembl.org/Homo_sapiens/Tools/VcftoPed), we entered the gene location, selected the Chinese Han population in Beijing as population, and downloaded the ped and info file for the SNPs of *CYP2C8*. We obtained 103 SNPs within *CYP2C8* from the database; (2) Then, we used Haploview software for quality control [minor allele frequency >5%, minor genotype >75%, *r* ^2^ < 0.8, and Hardy–Weinberg equilibrium (HWE) >0.05] to select tag-SNP. Finally, four SNPs (including rs1934953, rs1934951, rs2275620, and rs17110453) were selected for investigation. A DNA extraction kit was used for extracting the genomic DNA from peripheral blood samples. Agena Design software was used to design the PCR amplification primers. The detailed information of the primers in this study is listed in **Table 1**. SNP genotyping was determined using Agena MassARRAY iPLEX platform. Besides this, the data of genotypes was organized and analyzed by Agena Bioscience TYPER version 4.0 software.

**Table 1:** Primers used in this study.

SNP_ID	2nd-PCRP	1st-PCRP	UEP-DIR	UEP SEQ
rs1934953	ACGTTGGATGCTTGTTTCCTGTTCCAAGCC	ACGTTGGATGAGAGAGTGTATGACCAGAGC	F	AAGCCTGATATTCCATGA
rs1934951	ACGTTGGATGGTTGGAATTTACATGGCACC	ACGTTGGATGATGGGTGTTAAGAGTGGTGC	R	GGGGCTGGTAGAATTGCTATTT
rs2275620	ACGTTGGATGCATCTTGTGTTGTTAGAGGG	ACGTTGGATGCCCCAAGGTAAGCTTGTTTC	F	ccAACCAAACCAGCACTC
rs17110453	ACGTTGGATGACACTGATTTCCCTCAAGGT	ACGTTGGATGCTGTGATGATGGAGAAACAC	R	cgGATTTCCCTCAAGGTCATAAA

SNP, single-nucleotide polymorphisms; PCRP, polymerase chain reaction primer; UEP-DIR, unextended primer sequence direction; UEP SEQ, unextended mini-sequencing primer sequence.

1st-PCRP, first PCR primer; 2nd-PCRP, second PCR primer.

### Statistical analyses

All statistical tests in this study were two-sided and carried out with SPSS 22.0 software. The two-tailed *p*-value <0.05 was considered to have a statistical difference. *χ* ^2^ test and Student’s t-test were used to detect the statistical differences in age and sex between cases and controls, respectively. HWE in controls was determined by Fisher’s exact test. The impact of *CYP2C8* polymorphisms on the risk of bladder cancer was tested by logistic regression analysis under five genetic models (allele, dominant, codominant, log-additive, and recessive). We also investigated the association of stratification analyses. Odds ratios (ORs) and 95% confidence intervals (CIs) were calculated to check the associations. Finally, we explored the influence of SNP–SNP interactions on bladder cancer *via* multifaceted dimensionality reduction (MDR) analysis.

## Results

### Study participants

The distributions of demographic variables of bladder cancer and healthy individuals are shown in **Table 2**. The average age of the cases was 64.40 ± 10.99 years, and the average age of the controls was 63.92 ± 6.62 years. There was no statistical difference in age between the patients and controls (*p* = 0.549), while a significant difference in sex was observed between the two groups (*p* = 0.001).

**Table 2:** Characteristics of bladder cancer patients and healthy controls in this study.

Characteristics	Cases (*n* = 217)	Controls (*n* = 550)	*p*
Age, years (mean ± SD)^a^	64.40 ± 10.99	63.92 ± 6.62	0.549
>65	103 (47%)	197 (36%)	
≤65	114 (53%)	353 (64%)	
Gender^b^			**0.001**
Male	175 (81%)	379 (69%)	
Female	42 (19%)	171 (31%)	
Clinical stage			
I/II	68 (31%)		
III/IV	86 (40%)		
Missing	63 (29%)		

p <0.05 indicates statistical significance.

a The p-value was calculated by Student’s t-test.

b The p-value was calculated by χ^2^ test. The bold values mean statistically significant.

### The association of *CYP2C8* polymorphisms with the risk of bladder cancer

Four SNPs in the *CYP2C8* gene were successfully genotyped in the study. As presented in **Table 3**, all SNPs in the control group were in line with the HWE (all *p >*0.05). The effect of *CYP2C8* variants on bladder cancer was then evaluated with logistic regression analysis, as shown in **Table 4**. Our study demonstrated that rs1934951 (codominant model: OR 1.96, *p* = 2.67E-04; dominant model: OR 1.74, *p* = 0.002) and rs17110453 (codominant model: OR 1.89, *p* = 2.53E-04; dominant model: OR 1.63, *p* = 0.004; recessive model: OR 1.46, *p* = 0.013) were significantly associated with an increased susceptibility to bladder cancer. Conversely, rs1934953 (allele: OR 0.61, *p* = 2.70E-05; codominant model: OR 0.26, *p* = 1.20E-05; dominant model: OR 0.62, *p* = 0.005; recessive model: OR 0.31, *p* = 5.38E-05; log-additive: OR 0.58, *p* = 2.17E-05) and rs2275620 (codominant model: OR 0.40, *p* = 0.005; recessive model: OR 0.32, *p* = 1.41E-04) showed a protective effect on the risk of bladder cancer.

**Table 3:** Basic information and allele frequencies of *CYP2C8* SNPs.

SNP ID	Chromosome position	Role	Alleles (minor/major)	MAF		O (HET)	E (HET)	*p^a^*-HWE
				Case	Control			
rs1934953	chr10: 95037713	Intron	T/C	0.364	0.420	0.533	0.498	0.123
rs1934951	chr10: 95038791	Intron	T/C	0.294	0.313	0.486	0.475	0.590
rs2275620	chr10: 95042841	Intron	T/A	0.290	0.316	0.525	0.494	0.167
rs17110453	chr10: 95069772	3′UTR	C/A	0.297	0.339	0.433	0.451	0.345

p-values were calculated by exact test. p <0.05 indicates statistical significance.

SNP, single-nucleotide polymorphisms; MAF, minor allele frequency; HWE, Hardy–Weinberg equilibrium.

**Table 4:** Association analysis between *CYP2C8* SNPs and bladder cancer risk.

SNP ID	Model	Geno type	Case *N*	Control *N*	Without adjusted		With adjusted	
					OR (95% CI)	*p^a^*	OR (95% CI)	*p^b^*
rs1934953	Allele	C	282	585			1	
		T	152	515			0.61 (0.49–0.77)	**2.70E-05**
	Codominant	CC	80	146	1		1	
		CT	122	293	0.76 (0.54–1.07)	0.119	0.74 (0.53–1.05)	0.096
		TT	15	111	0.68 (0.46–0.99)	5.59E-06	0.26 (0.14–0.47)	**1.20E-05**
	Dominant	CC	80	146	1		1	
		TC-TT	137	403	0.62 (0.44–0.86)	0.005	0.62 (0.44–0.86)	**0.005**
	Recessive	CC-TC	202	439	1		1	
		TT	15	111	0.29 (0.17–0.52)	2.09E-05	0.31 (0.18–0.55)	**5.38E-05**
	Log-additive	–	–	–	0.58 (0.45–0.74)	1.19E-05	058 (0.45–0.75)	**2.17E-05**
rs1934951	Allele	C	253	670			1	
		T	181	424			1.13 (0.90–1.42)	0.288
	Codominant	CC	55	202	1		1	
		CT	143	266	1.97 (1.38–2.83)	2.19E-04	1.96 (1.37–2.82)	**2.67E-04**
		TT	19	79	0.88 (0.49–1.58)	0.677	0.94 (0.52–1.69)	0.82
	Dominant	CC	55	202	1		1	
		TC-TT	162	345	1.73 (1.21–2.45)	0.002	1.74 (1.22–2.48)	**0.002**
	Recessive	CC-TC	198	468	1		1	
		TT	19	79	0.57 (0.34–0.96)	0.036	0.60 (0.35–1.03)	0.064
	Log-additive	–	–	–	1.15 (0.90–1.46)	0.258	0.58 (0.92–1.51)	0.189
rs2275620	Allele	A	260	608			1	
		T	174	490			0.83 (0.66–1.04)	0.107
	Codominant	AA	57	160	1		1	
		AT	146	288	1.42 (0.99–2.04)	0.056	1.38 (0.96–1.99)	0.081
		TT	14	101	0.39 (0.21–0.73)	3.60E-03	0.40 (0.21–0.76)	**0.005**
	Dominant	AA	57	160	1		1	
		TA-TT	160	389	1.16 (0.81–1.65)	0.426	1.14 (0.80–1.62)	0.482
	Recessive	AA-TA	203	448	1		1	
		TT	14	101	0.31 (0.17–0.55)	6.84E-05	0.32 (0.18–0.58)	**1.41E-04**
	Log-additive	–	–	–	0.80 (0.63–1.03)	0.080	0.81 (0.63–1.04)	0.092
rs17110453	Allele	A	275	722			1	
		C	159	378			1.10 (0.88–1.39)	0.401
	Codominant	AA	71	242	1		1	
		AC	133	238	1.91 (1.36–2.67)	1.96E-04	1.89 (1.35–2.67)	**2.53E-04**
		CC	13	70	0.63 (0.33–1.21)	0.167	0.66 (0.34–1.27)	0.213
	Dominant	AA	71	242	1		1	
		CA-CC	146	308	1.62 (1.16–2.25)	0.004	1.63 (1.16–2.27)	**0.004**
	Recessive	AA-CA	204	480	1		1	
		CC	13	70	0.44 (0.24–0.81)	0.008	1.46 (0.25–0.85)	**0.013**
	Log-additive	–	–	–	1.11 (0.83–1.41)	0.386	1.13 (0.83–1.44)	0.334

p <0.05 indicates statistical significance.

CI, confidence interval; OR, odds ratio; SNP, single-nucleotide polymorphism.

a p‐values were calculated by logistic regression analysis without adjustment.

b p‐values were calculated by logistic regression analysis with adjustment for age and gender. The bold values mean statistically significant.

### Stratified analyses

The stratification of individuals according to their age (**Table 5**) indicated that rs1934951 (>65 years: CT *vs*. CC, OR 2.17, *p* = 0.009; TC-TT *vs*. CC, OR 1.82, *p* = 0.037; ≤65 years: CT *vs*. CC, OR 1.87, *p* = 0.016; TC-TT *vs*. CC, OR 1.74, *p* = 0.030, respectively) and rs17110453 (>65 years: AC *vs*. AA, OR 1.96, *p* = 0.016; ≤65 years: AC *vs*. AA, OR 1.93, *p* = 0.007; AC-CC vs AA, OR 1.64, *p* = 0.038) significantly increased the susceptibility to bladder cancer. rs1934953 was related to decreased susceptibility to bladder cancer in people aged >65 years (T *vs*. C, OR 0.62, *p* = 0.007; TT *vs*. CC, OR 0.29, *p* = 0.005; TT *vs*. CC-TC, OR 0.34, *p* = 0.006) and aged ≤65 years (T *vs*. C, OR 0.58, *p* = 6.24E-04; TT *vs*. CC, OR 0.25, *p* = 0.003; TT *vs*. CC-TC, OR 0.30, *p* = 0.008). Besides this, rs2275620 also had a protective effect on bladder cancer risk in individuals aged >65 years (TT *vs*. AA-TA, OR 0.44, *p* = 0.034) and aged ≤65 years (TT *vs*. AA-TA, OR 0.21, *p* = 0.004).

**Table 5:** Association of *CYP2C8* SNPs with the risk of bladder cancer stratified by age.

SNP	Model	Allele/genotype	Case	Control	OR (95% CI)	*p*	Case	Control	OR (95% CI)	*P*
**Age**			**>65**				**≤65**			
rs1934953	Allele	C	128	199	1		154	386	1	
		T	78	195	0.62 (0.44–0.88)	**0.007**	74	320	0.58 (0.42–0.79)	**6.24E-04**
	Codominant	CC	34	49	1		46	97	1	
		CT	60	101	0.79 (0.45–1.40)	0.425	62	192	0.74 (0.46–1.19)	0.212
		TT	9	47	0.29 (0.12–0.69)	**0.005**	6	64	0.25 (0.10–0.63)	**0.003**
	Dominant	CC	34	49	1		46	97	1	
		TC-TT	69	148	0.64 (0.37–1.11)	0.108	68	256	0.62 (0.39–1.00)	**0.048**
	Recessive	CC-TC	94	150	1		108	289	1	
		TT	9	47	0.34 (0.16–0.74)	**0.006**	6	64	0.30 (0.12–0.73)	**0.008**
	Log-additive	–	–	–	0.59 (0.40–0.87)	**0.008**	–	–	0.59 (0.41–0.84)	**0.004**
rs1934951	Allele	C	118	234	1		135	436	1	
		T	88	156	1.12 (0.79–1.58)	0.521	93	268	1.12 (0.83–1.52)	0.464
	Codominant	CC	25	72	1		30	130	1	
		CT	68	90	2.17 (1.21–3.86)	**0.009**	75	176	1.87 (1.13–3.11)	**0.016**
		TT	10	33	0.88 (0.37–2.10)	0.776	9	46	1.12 (0.48–2.62)	0.794
	Dominant	CC	25	72	1		30	130	1	
		TC-TT	78	123	1.82 (1.04–3.19)	**0.037**	84	222	1.74 (1.06–2.86)	**0.030**
	Recessive	CC-TC	93	162	1		123	276	1	
		TT	10	33	0.53 (0.25–1.15)	0.110	9	46	0.75 (0.34–1.62)	0.461
	Log-additive	–	–	–	1.13 (0.77–1.64)	0.538	–	–	1.27 (0.89–1.81)	0.196
rs2275620	Allele	A	117	209	1		143	399	1	
		T	89	183	0.87 (0.62–1.22)	0.417	85	307	0.77 (0.57–1.05)	0.099
	Codominant	AA	24	54	1		33	106	1	
		AT	69	101	1.47 (0.81–2.66)	0.207	77	187	1.32 (0.80–2.17)	0.280
		TT	10	41	0.58 (0.24–1.38)	0.218	4	60	0.25 (0.08–0.77)	**0.016**
	Dominant	AA	24	54	1		33	106	1	
		TA-TT	79	142	1.22 (0.68–2.18)	0.505	81	247	1.08 (0.66–1.77)	0.759
	Recessive	AA-TA	93	155	1		110	293	1	
		TT	10	41	0.44 (0.21–0.94)	**0.034**	4	60	0.21 (0.07–0.61)	**0.004**
	Log-additive	–	–	–	0.85 (0.58–1.26)	0.425	–	–	0.77 (0.54–1.11)	0.156
rs17110453	Allele	A	125	253	1		150	469	1	
		C	81	141	1.16 (0.82–1.65)	0.395	78	237	1.03 (0.75–1.41)	0.859
	Codominant	AA	30	85	1		41	157	1	
		AC	65	83	1.96 (1.13–3.39)	**0.016**	68	155	1.93 (1.19–3.12)	**0.007**
		CC	8	29	0.67 (0.27–1.69)	0.398	5	41	0.56 (0.20–1.55)	0.265
	Dominant	AA	30	85	1		41	157	1	
		CA-CC	73	112	1.62 (0.95–2.76)	0.074	73	196	1.64 (1.03–2.62)	**0.038**
	Recessive	AA-CA	95	168	1		109	312	1	
		CC	8	29	0.45 (0.19–1.05)	0.065	5	41	0.39 (0.15–1.03)	0.058
	Log-additive	–	–	–	1.07 (0.74–1.57)	0.714	–	–	1.14 (0.80–1.61)	0.467

p-values were calculated by logistic regression adjusted by age and gender. p <0.05 indicates statistical significance.The bold values mean statistically significant.

### The influence of SNP–SNP interactions on bladder cancer susceptibility

**Figure 1:**
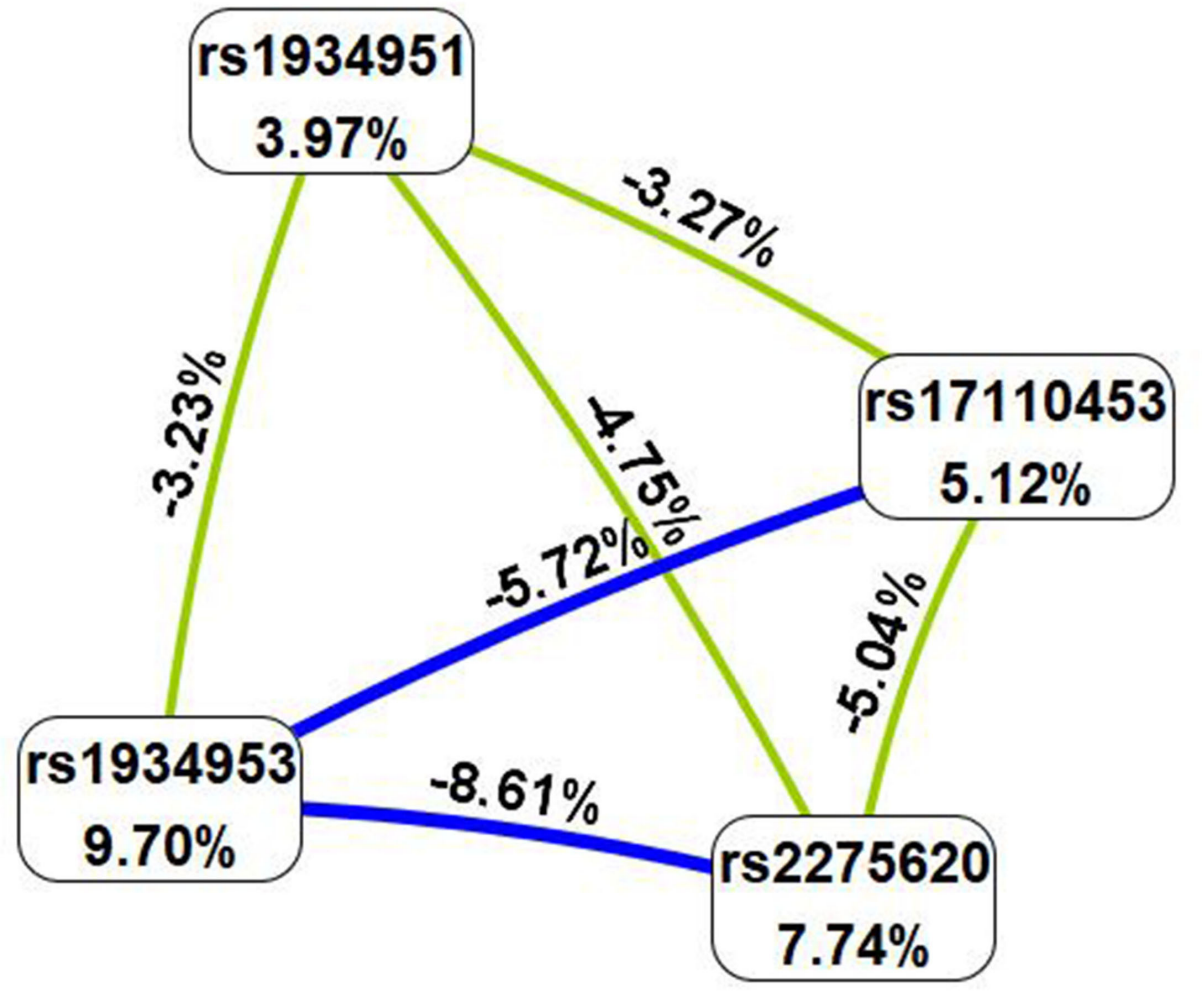
SNP–SNP interaction map. Values in nodes represent the information gains of individual attribute (main effects). Values between nodes are information gains of each pair of attributes (interaction effects). Blue and green with negative percent entropy indicate redundancy or independence.

MDR analysis was carried out to explore the correlation between SNP–SNP interactions and bladder cancer. The MDR method selects variables with attribute interactions on the basis of entropy measures for evaluating the information gain associated with attribute interactions. The patterns of entropy recapitulated the main and/or interaction effect of each pairwise combination of attributes. As shown in **Figure 1**, the interaction map with negative percent entropy represented the independence or redundancy of each pairwise combination of attributes (-8.61, -5.72, -5.04, -4.75, -3.27, and -3.23%, respectively, shown in blue and green), and there was a strong independence or redundancy between rs1934953 and rs2275620, with the information gain values of -8.61%. **Table 6** shows that the combination of rs1934953, rs1934951, rs2275620, and rs17110453 was the best model to predict bladder cancer (CVC = 10/10, testing accuracy = 0.6720, *p* < 0.0001). The best single-locus model was rs17110453 (CVC = 9/10, testing accuracy = 0.6083, *p* < 0.0001). The best two-locus model consisted of rs1934953 and rs1934951 (CVC = 6/10, testing accuracy = 0.6242, *p* < 0.0001). rs1934953, rs2275620, and rs17110453 formed the best three-locus model (CVC = 7/10, testing accuracy = 0.6561, *p* < 0.0001).

**Table 6:** Analysis of the SNP–SNP interaction models with the multifactor dimensionality reduction method.

Model	Training bal. acc.	Testing bal. acc.	CVC	OR (95% CI)	*p*
rs17110453	0.6246	0.6083	9/10	2.81 (1.77–4.46)	**<0.0001**
rs1934953, rs1934951	0.6798	0.6242	6/10	5.61 (3.29–9.59)	**<0.0001**
rs1934953, rs2275620, rs17110453	0.7162	0.6561	7/10	6.75 (4.06–11.23)	**<0.0001**
rs1934953, rs1934951, rs2275620, rs17110453	0.7272	0.6720	10/10	7.64 (4.56–12.77)	**<0.0001**

p-values were calculated by χ^2^ test. p <0.05 indicates statistical significance.

Bal. Acc., balanced accuracy; CVC, cross-validation consistently.The bold values mean statistically significant.

## Discussion

Genetic factors play a significant role in the development and progression of bladder cancer. In this study, we firstly examined the correlation of *CYP2C8* genetic variants with the risk of bladder cancer in the Chinese population. Our findings indicated that the SNPs in the *CYP2C8* gene were significantly related to bladder cancer susceptibility. Our research provides a new perspective for understanding the molecular mechanism of the correlation between genetic background and carcinogenesis in bladder cancer.

*CYP2C8* is located on chromosome 10q24. One study has shown that *CYP2C8* polymorphisms have a certain functional significance (24). Some studies have shown that functional polymorphisms that affect the expression or activity of the *CYP2C8* gene can significantly increase the susceptibility to bladder cancer (25, 26). Various studies have been conducted on the association of *CYP2C8* polymorphisms with human cancers—for example, Golpar *et al*. have reported that rs1058930 of *CYP2C8* could significantly increase breast cancer risk (27). It has been reported that *CYP2C8* polymorphisms can significantly change the imatinib metabolism in patients with leukemia through both gain- and loss-of-function mechanism (28). Another study has indicated that *CYP2C8* variations can influence ovarian cancer risk (29). However, the relationship between *CYP2C8* polymorphisms and bladder cancer risk has not been reported. In our study, we found that rs1934951 and rs17110453 in *CYP2C8* significantly increased the risk of bladder cancer. rs1934953 and rs2275620 were related to a reduced risk of bladder cancer. The stratification analysis suggested that the impact of *CYP2C8* polymorphisms on bladder cancer susceptibility may be independent of age. This result is contrary to the fact that age is a risk factor for bladder cancer, which may be caused by the small sample size.

The study of SNP–SNP interactions is helpful to find more risk factors for bladder cancer. Interestingly, we observed that there was a strong independence or redundancy between rs1934953 and rs2275620. In addition, the combination of rs1934953, rs1934951, rs2275620, and rs17110453 was the best model to predict bladder cancer.

Some limitations in our present study should be noted. First, the sample size is relatively small, and we will further verify our conclusions by expanding the sample size in the future. Second, the associations stratified by smoking status and clinical stage were not detected on account of the limited information obtained from the medical records. Third, although we determined the impact of *CYP2C8* polymorphisms on bladder cancer risk, the molecular mechanism of *CYP2C8* polymorphisms affecting bladder cancer has not been investigated in this work. In spite of the abovementioned shortage, our study is the first to examine the association of *CYP2C8* polymorphisms with bladder cancer risk, which may give a new biomarker for the diagnosis or prevention of bladder cancer in the Chinese population.

## Data availability statement

The original contributions presented in the study are publicly available. This data can be found here: https://doi.org/10.5281/zenodo.7074453.

## Ethics statement

The studies involving human participants were reviewed and approved by the Shaanxi Provincial Cancer Hospital and the 1964 Helsinki Declaration. The patients/participants provided their written informed consent to participate in this study.

## Author contributions

WQ and JZ were responsible for the study design. WQ processed the data and wrote the manuscript. YC and JL recruited the study participants. FZ contributed to the primer design and data analysis. JZ revised the paper. All authors contributed to the article and approved the submitted version.

## Funding

This study was supported by the Key Research & Development Projects in Shaanxi Province (grant no. 2022SF-465 to W. Qu), the Science Foundation of Shaanxi Provincial People’s Hospital (grant no. 2021YJY-30 to J. Zhou) and the Shaanxi Provincial Natural Science Foundation (grant no. 2020JM-657 to J. Zhou).

## Acknowledgments

We thank all participants in this study. We also thank the Shaanxi Provincial Cancer Hospital for their helping with sample collections.

## Conflict of interest

The authors declare that the research was conducted in the absence of any commercial or financial relationships that could be construed as a potential conflict of interest.

## Publisher’s note

All claims expressed in this article are solely those of the authors and do not necessarily represent those of their affiliated organizations, or those of the publisher, the editors and the reviewers. Any product that may be evaluated in this article, or claim that may be made by its manufacturer, is not guaranteed or endorsed by the publisher.
